# Prezygotic reproductive isolation between *Saccharomyces cerevisiae *and *Saccharomyces paradoxus*

**DOI:** 10.1186/1471-2148-8-1

**Published:** 2008-01-07

**Authors:** Calum J Maclean, Duncan Greig

**Affiliations:** 1 The Galton Laboratory, University College London, 4 Stephenson Way, London, NW1 2HE, UK

## Abstract

**Background:**

Matings between different *Saccharomyces *sensu stricto yeast species produce sexually sterile hybrids, so individuals should avoid mating with other species. Any mechanism that reduces the frequency of interspecific matings will confer a selective advantage. Here we test the ability of two closely-related *Saccharomyces *sensu stricto species to select their own species as mates and avoid hybridisation.

**Results:**

We set up mate choice tests, using five independently isolated pairs of species, in which individual germinating spores were presented with the opportunity to mate either with a germinating spore of their own species or with a germinating spore of the other species. For all five strain pairs, whether a *S. cerevisiae *or *S. paradoxus *occupies the role of "chooser" strain, the level of hybridisation that is observed between the two species is significantly lower than would be expected if mates were selected at random. We also show that, overall, *S. cerevisiae *exhibited a stronger own-species preference than *S. paradoxus*.

**Conclusion:**

Prezygotic reproductive isolation is well known in higher organisms but has been largely overlooked in yeast, an important model microbe. Here we present the first report of prezygotic reproductive isolation in *Saccharomyces*. Prezygotic reproductive isolation may be important in yeast speciation or yeast species cohesion, and may have evolved to prevent wasted matings between different species. Whilst yeast has long been used as a genetic model system, little is known about yeast in the wild. Our work sheds light on an interesting aspect of yeast natural behaviour: their ability to avoid costly interspecific matings.

## Background

The biological species concept defines a species as an interbreeding group that is reproductively isolated from other such groups [1]. Species are isolated by barriers that either prevent fertilisation between species (prezygotic barriers) or those that allow fertilisation but make the resulting hybrid sterile or inviable (postzygotic barriers) [2] (for a review see [3]).

Mating in yeast occurs through the fusion of haploid gametes. When starved, diploid *Saccharomyces *yeast cells produce haploid spores by meiosis. Each diploid cell produces four dormant and resilient haploid spores, two spores of each mating type (**a **and **α**). When nutrients become available again the spores germinate to become metabolically active gametes. Gametes of both mating types produce attractive pheromones used to signal to the other mating type. Gametes of different mating-types fuse, producing diploid zygotes that can reproduce asexually by mitosis until nutrients are exhausted again [4,5].

*Saccharomyces *sensu stricto species are postzygotically isolated. Diploid F1 hybrids are formed by fusion of gametes from different species. These hybrids can reproduce asexually by mitosis, but spores produced by meiosis are inviable, failing to germinate and form gametes [6]. Thus F1 hybrids are viable but sexually sterile. Several recent investigations have examined possible causes of this hybrid sterility, and concluded that sequence and chromosomal differences between the species are major contributors [7-9]. Two *Saccharomyces *species, *S. cerevisiae *and *S. paradoxus*, have been found to occupy the same natural habitat (oak trees and associated soils) [10], providing the opportunity for hybridisation. Kuehne et al [11] have recently shown that the North American and Eurasian *S. paradoxus *isolates represent two distinct groups. Within each group the strains are highly related (indicating a large breeding population) and have distributions spanning their respective land masses [11]. The population structure of *S. cerevisiae *is not so clear, perhaps because human domestication of the species overshadows their natural biogeography [12]. Yeast hybrids can be formed in the laboratory but wild F1 hybrids, containing a full genome from both *S. paradoxus *and *S. cerevisiae*, have not been described [12]. Several reports have, however, shown introgression of genes between the two species, indicating that interspecific mating can occur in the wild [12-14]. Given that hybrids are sexually sterile, the ability to avoid hybridisation may be favoured by natural selection.

In a recent paper Murphy et al. [15] failed to find prezygotic reproductive isolation between species from sympatric natural populations of *S. cerevisiae *and *S. paradoxus*. Murphy et al. [15] assayed species recognition using individual mate choice trials: a single vegetative haploid cell of known mating type was placed in contact with a conspecific and a heterospecific vegetative cell of the opposite mating type. The results showed that *S. cerevisiae *cells mated with other *S. cerevisiae *cells more often than they mated with *S. paradoxus *cells, as expected if a prezygotic barrier existed. But, surprisingly, Murphy et al. [15] found that *S. paradoxus *cells mated with *S. cerevisiae *cells (forming sterile hybrids) more often than they mated with other *S. paradoxus *cells. They explained this result by the observation that the mating propensity (the tendency or readiness to mate) of *S. cerevisiae *gametes was higher than that of *S. paradoxus *gametes and therefore focal cells of either species were more likely to be able to mate with the more willing *S. cerevisiae *gametes, regardless of whether or not a hybrid zygote was produced. This difference in mating propensity confounded the quantification of prezygotic isolation between the species, and Murphy et al. [15] were unable to detect prezygotic reproductive isolation. However they proposed that differences in mating kinetics could potentially provide a prezygotic isolation barrier, because fast maters would tend to mate with fast maters, and slow maters with slow maters. Such a barrier can evolve readily in the laboratory under artificial selection against mating between genetically marked strains of the same species [16].

Although very little is known about yeast life history in nature, it is likely that most mating occurs immediately after germination, usually between members of the same tetrad, and without any haploid cell division [17]. Because wild yeast are naturally homothallic (see for example [18] and [19]), and unfertilized gametes can switch mating type after dividing to enable them to fuse with their daughter cells, even isolated single spores should yield diploid, not haploid cultures [4]. Murphy et al. [15] prevented their strains from switching mating type in culture by knocking out their *HO *genes with drug-resistance markers, allowing the culture of clones of vegetative haploid gametes. However such clones of unfertilised gametes are not thought to occur in natural strains with intact *HO *genes. Unfertilised gametes exist only rarely and transiently, after spore germination but before fertilisation by either a neighbouring germinated spore or mating-type switched clone-mate. Thus the potential for hybridization in nature is highest when spores from different species happen to be in contact at the time of germination. Such close contact between spores from different species might occur if they occupy the same habitat (e.g. the surface of oak trees), or if they are brought together in the digestive tracts of species that eat yeast [20]. Yeast-feeding insects, such as *Drosophila*, completely digest vegetative yeast cells, but yeast spores are not digested and are passed through the gut unharmed and ready to germinate [21]. Therefore prezygotic reproductive isolation is likely to involve species differences in germination conditions or timing as well as in gamete fusion.

Here we present the results of mate choice assays using wild type homothallic (*HO*) single spores of five *S. cerevisiae *and five *S. paradoxus *isolates from natural populations. All pairings were made between strains isolated from the same continent (either North America or Eurasia). Strains used in three of the pairings were both isolated from the same small woodland area and can be considered sympatric [18,11] (see Methods for full strain details).

## Results

### Both species avoid hybridisation

**Figure 1:**
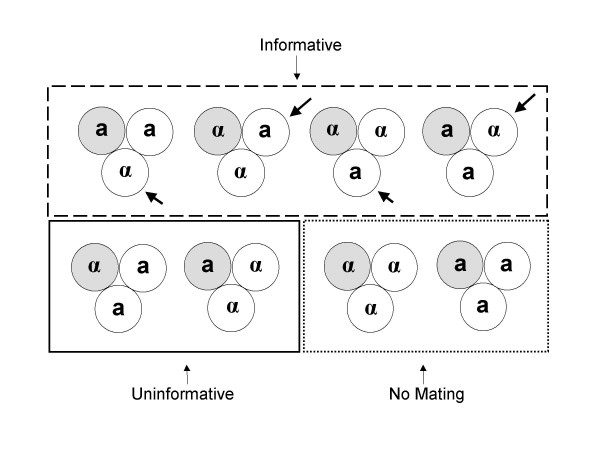
**Mate choice pairing possibilities**. The eight possible combinations when a spore of one species is placed against two spores from another species. Grey and white circles indicate species, arrows indicate cells with a choice of mates, and **a **and **α **indicate the mating types of the spores.

Individual mate choice assays were conducted by placing two spores of one species (the chooser strain) in contact with a single spore from the other species. All spores were taken from different tetrads to ensure mating types were sampled randomly. Since it is impossible to determine the mating types of spores before germination, deviations from random mating were calculated based on probability (see Methods and Figure 1). Hybrid and non-hybrid zygotes were identified using species specific PCR. Mate choice trials were carried out using five independent pairs of *S. cerevisiae *and *S. paradoxus *strains (Table 1).

**Table 1:** Strain table

Pairing	Known as	Strain	Species	Isolated From	Location	Ref.
1	*Sc1*	Y55	*S. cerevisiae*	Wine Grape	France ^a^	[14]
	*Sp1*	N-17	*S. paradoxus*	Oak tree	Tatarstan, Russia ^b^	[29]
2	*Sc2*	SK1	*S. cerevisiae*	Soil (Lab strain)	USA (exact location unknown) ^c^	[14]
	*Sp2*	YPS138	*S. paradoxus*	Soil beneath oak tree	Tyler Arboretum Media, PA, USA ^d^	[30]
3	*Sc3*	YPS128	*S. cerevisiae*	Soil beneath oak tree	Tyler Arboretum Media, PA, USA ^d^	[30]
	*Sp3*	YPS145	*S. paradoxus*	Soil beneath oak tree	Tyler Arboretum Media, PA, USA ^d^	[30]
4	*Sc4*	YPS681	*S. cerevisiae*	Oak tree	Buck Hill Falls, PA, USA ^e^	[15]
	*Sp4*	YPS664	*S. paradoxus*	Oak tree	Buck Hill Falls, PA, USA ^e^	[15]
5	*Sc5*	YPS670	*S. cerevisiae*	Oak tree	Buck Hill Falls, PA, USA ^e^	[15]
	*Sp5*	YPS646	*S. paradoxus*	Oak tree	Buck Hill Falls, PA, USA ^e^	[15]

Table showing the strains used in each of the 5 species pairs.

For the purpose of our analysis, we considered pairs 1 and 2 to be allopatric and pairs 3, 4, and 5 to be sympatric.

^a ^– Exact time of isolation unknown but believed to be between 1930 and 1960.

^b ^– Exact time of isolation unknown – first published reference is from 1988 [31]

^c ^– Exact time of isolation unknown – first published reference from 1974 [32]

^d ^– Isolated in July 1999

^e ^– Isolated in 2000

**Figure 2:**
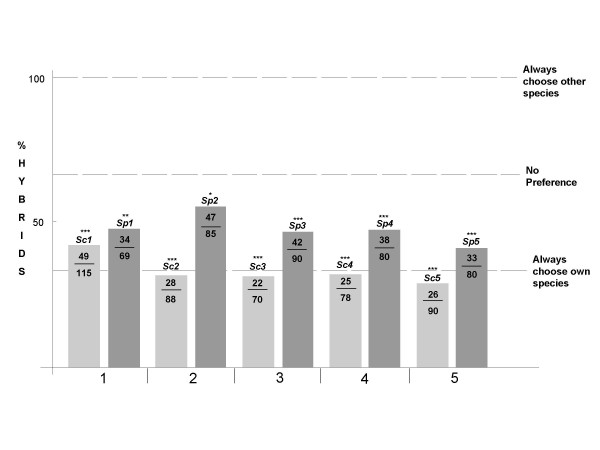
**Graphs of % hybrid matings for each pairing**. Bar chart showing the percentage of matings that resulted in hybrid zygotes for the five species pairs. For each pair, the light grey bar represents the result when *S. cerevisiae *(*Sc*) chose and the dark grey bar represents the result when *S. paradoxus *(*Sp*) chose. On each bar the numbers are the number of hybrid zygotes formed over the total number analysed. Dashed lines indicate the proportions of hybrids that would be expected if the chooser always mated with its own species (33.33%), had no preference (66.67%) and always mated with the other species (100%). All strains formed significantly fewer hybrids than would be expected by chance (*** = p < 0.001, ** = p < 0.01. * = p < 0.05). (For full dataset see Table 2.)

Figure 2 shows the proportion of matings that resulted in hybrid zygotes for each of the five independent species pairs. If mating was random with respect to species, then on average 2/3 (66.67%) of the zygotes formed would be hybrids (see Methods). Instead, we found that for every pair, whether *S. cerevisiae *or *S. paradoxus *occupied the role of chooser, significantly fewer hybrid zygotes were formed than expected by random mating (full data in Figure 2 and Table 2).

**Table 2:** Mate choice information table

Pair	"chooser" strain	Total trials as "chooser"	Total zygotes formed	Observed number of hybrid zygotes	Expected number of hybrid zygotes^*a*^	Hybrid zygote χ^2 ^(p value)^*b*^	Always own species χ^2 ^(p value)^*c*^	Mating propensity^*d*^	Mating Prospensity χ^2 ^(p value)
1	*Sc1*	300	115	49	76.67	28.89 (<0.001)	4.882943 (0.0271)	51.11	1.035 (0.3089)
	*Sp1*	206	69	34	46	8.63 (0.0033)	8.628882 (0.0033)	44.66	
2	*Sc2*	270	88	28	58.67	31.82 (<0.001)	0.035264 (0.8510)	43.46	2.212 (0.1369)
	*Sp2*	220	85	47	56.67	4.45 (0.0349)	19.4555 (<0.0001)	51.51	
3	*Sc3*	250	70	22	46.67	31.42 (<0.001)	0.044643 (0.8327)	37.33	0.164 (0.6857)
	*Sp3*	344	90	42	60	15.31 (<0.001)	7.8125 (0.0052)	34.88	
4	*Sc4*	210	78	25	52	40.51 (<0.001)	0.014423 (0.9044)	49.52	0.0046 (0.9462)
	*Sp4*	220	80	38	53.33	12.38 (<0.001)	7.876563 (0.005)	48.48	
5	*Sc5*	240	90	26	60	56.13 (<0.001)	0.877974 (0.3488)	50.00	2.229 (0.1355)
	*Sp5*	260	80	33	53.33	22.13 (<0.001)	2.626563 (0.1051)	41.03	

Table showing the raw data obtained from the mate choice trials as well as the hybrid zygote and mating propensity χ^2 ^values.

^*a *^– The expected number of hybrid zygotes if no mating preference exists between the two species (2/3 total – see Methods)

^*b *^– χ^2 ^test on the number of hybrid zygotes observed compared to the number expected if there is no mate preference (Yates corrected) (see also Figure 2).

^*c *^– χ^2 ^test on the number of hybrid zygotes observed compared to the 1/3 expected under the "always choose own species" model. (see Figures 1 and Methods)

^*d *^– Percentage of mating trials producing zygotes after correcting for the 25% of trials that cannot form zygotes because all three spores are the same mating type (see Methods).).

^*e *^– χ^2 ^test for the effect of species in pair on mating propensity (Yates corrected).

### Both species in each pair had similar mating propensities

Many trials did not result in a zygote, either because all three spores in a trial were the same mating type (this will occur on average in 25% of trials – see Figure 1) or because mating does not always occur even when trials do contain gametes of both mating types. Differences in mating propensity between the species in each pair would mean that a higher proportion of trials would result in zygotes when the species with a high mating propensity was "chooser" than when the species with a low mating propensity was chooser. For each pair, we found no significant difference between the number of zygotes formed when either strain was chooser (Table 2).

### *S. cerevisiae *is choosier than *S. paradoxus*

Do spores always choose to mate with a member of their own species, if available? If hybrids only form when there is no mate of the same species available, then on average only 1/3 of zygotes formed would be expected to be hybrids (Figure 1). We tested whether there was signification deviation from this expectation (Table 2). Only one of the five *S. cerevisiae *strains (*Sc1*) produced a significantly higher proportion of hybrid zygotes than the 1/3 expected if a *S. cerevisiae *strain always chose to mate with a member of its own species. In contrast four of the five *S. paradoxus *strains (*Sp1, Sp2, Sp3 and Sp4*) produced a significantly higher number of hybrid zygote than the 1/3 predicted under the "always choose own species" model. We also noted that, in all five pairs, fewer hybrid zygotes were formed when *S. cerevisiae *was chooser than when *S. paradoxus *was chooser. This suggested prezygotic isolation was stronger when *S. cerevisiae *was the chooser than when *S. paradoxus *was chooser.

We wanted to determine the effect of two factors on the strength of prezygotic reproductive isolation: the species choosing, and whether the pair of species were isolated in allopatry or sympatry (see Table 1). To do this we performed a joint analysis by means of a Generalised Linear Model (GLM). To accommodate the binary structure of the data (hybrid mating vs. non-hybrid mating) we carried out a binomial GLM with logit link function. We included the factors 'species' (*S. cerevisiae *or *S. paradoxus*), 'locality' (sympatric or allopatric) and their interaction. The analysis was performed using the statistical package R (version 2.4.1, R Development Core Team 2007) [22]. The GLM showed that across sympatric and allopatric matings, species differ significantly in the level of pre-zygotic isolation ('species' term, p = < 0.0001). Thus, *S. cerevisiae *is choosier than *S. paradoxus*. In addition, the data showed that across species, prezygotic isolation tended to be stronger in allopatric as compared to sympatric matings. However, this effect was not statistically significant ('locality' term, p = 0.07). Finally, the analysis showed that both species were similar in the change in discrimination between sympatric and allopatric individuals of the other species (interaction term, p = 0.93).

## Discussion

### Closely related *Saccharomyces *species show prezygotic reproductive isolation

We have demonstrated that the closely related species *S. cerevisiae *and *S. paradoxus *show lower levels of interspecific hybridisation than would be expected if mating between the two were random with respect to species. To our knowledge this is the first time that prezygotic reproductive isolation has been demonstrated between *Saccharomyces *yeast species. A previous report by Murphy et al [15] using similar mate choice trials showed that when *S. cerevisiae *occupied the role of chooser interspecific mating was lower than expected by random mating. However Murphy et al [15] did not find prezygotic isolation because the result was reversed when *S. paradoxus *was chooser – when offered a choice *S. paradoxus *preferentially mated with *S. cerevisiae *individuals, producing more hybrids than expected by chance. This effect was caused by differences in mating propensity – *S. cerevisiae *haploid vegetative cells were both faster maters and more likely to mate than *S. paradoxus *cells. Thus mating tended to occur with the partner that was more willing to mate, regardless of its species. This meant that any prezygotic isolation was undetectable because it was obscured by the large difference in mating propensity.

The protocol of Murphy et al [15] used strains that had been genetically modified to grow as stable clones of vegetative gametes. This meant that only the fusion element of the mating process was tested. In nature, yeast gametes probably only exist immediately after spore germination, so we used spores, rather than vegetatively-growing gametes, in our assay, so that the whole mating process could be tested. Murphy et al [15] kindly provided us with samples of the ancestors of their strains, and in our assay we were able to detect significant prezygotic reproductive isolation between them (pairings 4 and 5), as well as between species in the other three pairs tested (pairings 1, 2, and 3). Our results can only be explained by the presence of prezygotic reproductive isolation.

Nevertheless we were still interested in whether the strength (but not the direction) of the measured prezygotic isolation was confounded by differences in mating propensity. Unlike Murphy et al [15], we detected no differences in mating propensity between the strains in each pair, but we had limited power to detect small differences. We did, however, determine that hybrids were formed less often when *S. cerevisiae *was chooser than when *S. paradoxus *was chooser. This observation could be caused by *S. cerevisiae *having stronger preference for members of its own species than *S. paradoxus*., i.e. *S. cerevisiae *is choosier. Mating with the wrong species is equally bad for either species, so it is not immediately obvious how such a difference in choosiness might evolve. One possibility is that the barrier evolved in *S. cerevisiae *to prevent mating with another yeast species more frequently encountered than *S. paradoxus *(the barrier not providing complete prezygotic reproductive isolation between the two species studied here). However, another explanation, consistent with Murphy et al. [15], is that the difference is caused by a greater mating propensity in *S. cerevisiae *than in *S. paradoxus*, perhaps reflecting some difference in the evolution of these two species.

How strong is prezygotic reproductive isolation in yeast? Whilst hybrids form readily when no member of the same species is present, our results show that many strains (especially *S. cerevisiae*) have near perfect discrimination when given choice of species, and hybrids only occur in the 33% of matings in which no choice is available (see Fig 1 and Results). Clearly, this ability could be important for yeast in its natural environment. It is impossible, given our current lack of knowledge of wild yeast ecology, to say how often the situation of only three spores being in isolated contact (as in our mate trials) occurs in its natural environment. Due to the nature of the yeast tetrad it is highly likely that a gamete will usually find itself in close proximity to another gamete originating from the same tetrad. But the digestion of yeast tetrads by insect vectors releases spores from their tetrads, increasing inter-tetrad mating [21]. Insects that feed on different yeast species are therefore likely to increase the possibility of hybridisation between different yeast species [20]. These mate choice trials have allowed us to demonstrate that mating behaviour can reduce hybridisation in *Saccharomyces *species.

One caveat that must be noted is that we have only looked into the level of hybridisation between *S. cerevisiae *and *S. paradoxus *isolates but not between strains of the same species. It is possible that variation in the traits that result in the prezygotic isolation between species may also result in varying levels of hybridisation between different isolates of the same species. Within species variation would be an interesting avenue for further investigation, especially between genetically distinct strains such as the geographically isolated *S. paradoxus *"groups" identified by Kuehne et al [11] and Koufopanou et al [23].

### Did prezygotic reproductive isolation evolve by direct selection?

Though we have demonstrated that these two species show reduced levels of hybridisation we are not able to say how or why it evolved. There are two possibilities. The first is that natural selection has acted directly to reduce the costly formation of sterile hybrids. The second is that the prezygotic isolation is an indirect consequence of evolution, whether by selection on another trait, or by genetic drift, that happens to result in reduced hybridisation. A laboratory study has already shown that prezygotic isolation can evolve quickly between *Saccharomyces *in direct response to selection against hybrids [16]. We also know that the two species are likely to encounter each other in nature: strains of these two species can be isolated from the same trees [10,18] and the two species exhibit very similar phenotypic profiles [24].

One way to address whether prezygotic isolation evolved by direct selection is to compare the strength of isolation in sympatric and allopatric species pairs. Sympatric pairs are likely to interact more frequently than allopatric pairs, so if prezygotic reproductive isolation evolved to reinforce the species barrier it should be stronger in sympatric pairs than in allopatric pairs. Coyne and Orr have shown this to be true for a large number of *Drosophila *species pairs [[25,26], for review see [3]]. In our assay we found that prezygotic reproductive isolation was stronger in the three sympatric species pairs than in the two allopatric species pairs; however the difference was not statistically significant. But with only five pairs we have little statistical power and we anticipate that a larger study may well find a strong effect of sympatry on prezygotic reproductive isolation. Further, we note that the members of the two pairs we designated as allopatric do share the same continent, and recent work has shown that yeast populations inhabiting the same continent recombine freely [11,23]. Dispersal between continents appears to be very low, so it would be very interesting to measure prezygotic reproductive isolation (if any) between yeast species isolated from different continents.

### Possible prezygotic reproductive isolation barriers in yeast

There has been considerable debate over whether postzygotic or prezygotic reproductive isolation barriers are most important in maintaining distinct species [3]. As prezygotic barriers act earlier the general consensus is that they represent a stronger barrier to gene flow between populations. If no mating actually occurs in the first place genes cannot be exchanged between species [3]. In nature species pairs often exhibit both prezygotic and postzygotic barriers which over time have accumulated to reproductively isolate the populations. It is however extremely hard to determine which barrier evolved first to start the speciation process [3].

The *Saccharomyces *sensu stricto yeasts are known to be strongly postzygotically isolated from one another [7-9]. The prezygotic reproductive isolation identified here could represent a "work in progress", with the two species still undergoing selection and evolution towards less "leaky" barriers to hybridisation.

Several parts of the yeast life cycle can possibly act as prezygotic barriers to stop, or reduce, hybridisation. One possible barrier that is analogous to mate selection mechanisms in many higher organisms is pheromone recognition [3]. If each *Saccharomyces *species is able to identify members of its own species by the pheromone it produces, avoiding hybridisation may be possible. This however seems unlikely to be the case as the peptide sequence of the pheromone is conserved across the *Saccharomyces *sensu stricto species [27]. Differences in mating kinetics have been previously highlighted as a possible premating barrier between *Saccharomyces *species. Leu et al [16] showed experimentally that *S. cerevisiae *could evolve to avoid mating with strains that would generate lethal combinations of genetic markers in the progeny. The reduction in harmful matings evolved because of changes in the speed of mating, with faster maters mating with other compatible fast maters, slow maters mating with other compatible slow maters, thus reducing harmful mating. Murphy et al [15] proposed that a similar mechanism might work for wild yeast. They found that *S. cerevisiae *strains had a higher mating propensity than the *S. paradoxus *strains used in their experiments. They postulated that as *S. cerevisiae *was more willing to mate and did so quicker it would be possible that in certain natural situations all the fast and willing maters of one species could mate together and all the slow and unwilling maters could mate together, reducing the rate of hybrid formation.

The spores that we used, unlike vegetative haploid cells, are dormant and do not produce pheromone making them effectively invisible to gametes actively seeking a mate. Vegetative yeast gametes up regulate the production of pheromone early in the mating response. As yeast select partners on the strength of the pheromone signal they produce, *S. paradoxus *is more likely to choose the displaying *S. cerevisiae *as a mate (which being faster up regulates pheromone quicker) [5]. This explains the asymmetric mating preference observed by Murphy et al [15] in their vegetative cell assays. When spores are used, if one species germinates quickly and begins to mate before the other species has germinated hybridisation can be reduced. For example if *S. cerevisiae *is a faster germinator than *S. paradoxus *in a *S. paradoxus *"choosing" mate choice trial the *S. cerevisiae *spore germinates first and will not sense any pheromone. The now metabolically active *S. cerevisiae *cell will enter the cell cycle and begin vegetative asexual growth, preventing it from mating until it has divided (~2 h). If, during this time, the *S. paradoxus *cells germinate, they will be more likely to mate with their own species. As the majority of wild yeast are homothallic this may be particularly important because an unmated fast-germinating individual is also likely to undergo mating-type switching and subsequently mate with its own daughter cell, eliminating it from the pool of potential mates. We propose, therefore, that spore germination is an important prezygotic barrier that has previously been overlooked in yeast.

Due to the length of time speciation takes to evolve between species it is likely that, through selection, many isolating barriers can evolve simultaneously [3]. Single isolating barriers can be "leaky" allowing for some hybridisation between populations, whereas multiple prezygotic barriers working in unison can act to reduce hybridisation [3]. A combination of isolating barriers may be involved in the observed prezygotic reproductive isolation between *Saccharomyces *species. By developing the methods outlined here as well as those previously reported by Murphy et al [15] it may be possible to tease apart the relative importance of all parts of the *Saccharomyces *life cycle as they affect reproductive isolation.

## Conclusion

We have shown for the first time that the two closely related (and often sympatric) yeast species *S. cerevisiae *and *S. paradoxus *are prezygotically reproductively isolated. Because hybrids are sexually sterile, the ability to mate with the correct species could be an adaptation.

Our results contrast with those of an earlier study [15] that failed to detect prezygotic isolation between vegetative gametes from different species. Mating in wild yeast is most likely to take place between newly germinated spores, so differences in germination may allow the evolution of prezygotic isolation barriers. With greater knowledge of yeast life history, in particular exactly when in the life cycle mating typically occurs, we will be able to more fully understand the contribution of differences in germination and mating kinetics to reproductive isolation in the wild. Clearly it is desirable that further experiments be carried out to expand our knowledge of this important model organism in its natural environment.

## Methods

### Strains, media and growth conditions

A list of the strains used in this work can be found in Table 1. All strains were wild-type and unmodified from their original ancestral type. Strains were sporulated on potassium acetate plates (2% potassium acetate, 0.22% yeast extract, 0.05% glucose, 0.087% complete amino acid mix, 2.5% agar) for 4 days at 25°C. Mate choice assays were conducted on YEPD plates (1% yeast extract, 2% bactopeptone, 2% glucose, 2.5% agar) at 30°C.

### Mate choice assays

The ascus containing the yeast spores was digested using a standard zymolyase protocol [28]. Using a tetrad dissection microscope, two spores of the chooser species were taken from separate tetrads and placed in contact with each other and a third spore of the other species on a YEPD plate (see Fig. 1). This allowed the chooser strain the possibility of mating with a member of its own species or another species. All spores used came from different tetrads.

After incubation, unmated individuals were removed and zygotes left in place. If the removed individual was found to have been inviable the test was ignored because no choice of mate was possible. Two forms of triad were used: one in which the *S. cerevisiae *strain was chooser and the other with *S. paradoxus *as chooser. This allowed the level of reproductive isolation of both species to be investigated.

8 mating type combinations are possible when two spores from one species are paired with one from another (Fig. 1). Of these 8 possibilities, 4 can provide information on mate choice between *Saccharomyces *species (dashed box). These four contain both an **a **and **α **spore from the chooser species and a spore of the other species of either mating type. When this occurs a chooser strain cell has a choice of mate. The remaining possible combinations provide no useful information. Two (solid box) represent triads where mating occurs but there is no possibility for mate choice between species. Only hybrids can be formed by mating within such triads. The remaining two possible combinations (dotted box) are those where all individuals within a triad are of the same mating type and unable to mate.

If there is no preferential mating between the species, 2/3 of the zygotes will be hybrid. This is because 1/3 of all matings will always produce hybrids (solid box, Fig. 1) and if there is no preference 1/2 of the informative triads (2/3 – dashed box, Fig. 1) will produce hybrid zygotes (1/3 + (1/2 × 2/3) = 2/3). If, when offered choice, a spore always mates with its own species 1/3 of zygotes will be hybrid. Alternatively, if when presented with a choice of mate a cell always chooses to mate with a member of another species, all zygotes will be hybrid. Prezygotic isolation can therefore be measured by deviation from the proportion of interspecific matings expected if no preference exists (2/3).

### DNA extraction and PCR identification of hybrids

Matings are identified as being interspecific or intraspecific by species specific PCR. DNA was extracted from the colonies formed by each mating using a glass bead method [28].

Species specific primers were designed that only produce an amplicon if the genome of a particular species is present as a template. The primers were designed by aligning the reference genomes for the two species using the fungal alignment viewer provided by the Saccharomyces Genome Database [27]. Different amplicon lengths were used for each species. *S. cerevisiae *primers produced an amplicon of approximately 500 bp whilst those for *S. paradoxus *amplified approximately 300 bp of DNA. This simple design feature allowed for the quick and easy determination of hybrids and allowed us to control for the accidental use of an incorrect primer by contamination. Two sets of species specific PCR primers that amplify a different region of the particular species genome were used for each zygote to allow confirmation of results. Some species specific primers did not work with some strains of the correct species, presumably because the sequence at a primer site was polymorphic. New primers were designed in these cases. See Additional file 1: PCR primer sequences for the sequences of the primers and the strain pairs with which they were used.

## Authors' contributions

CJM developed the assay, carried out the experiment and analysed the data. DG designed and supervised the experiment. Both authors wrote the paper.

## Supplementary Material

Additional file 1 Table 1: PCR primer sequence table. Contains the sequences of the species specific primers used to identify hybrid and non-hybrid matings. Click here for file
