# Supplementary material for: Prezygotic reproductive isolation between Saccharomyces cerevisiae and Saccharomyces paradoxus
Source: BMC Evol Biol. 2008 Jan 7;8:1. doi: 10.1186/1471-2148-8-1 (PMC2249576; doi:10.1186/1471-2148-8-1)
Supplement: Additional file 1 — Table 1: PCR primer sequence table. Contains the sequences of the species specific primers used to identify hybrid and non-hybrid matings. [file 1471-2148-8-1-S1.doc]

## Table 1 – PCR primer sequence table

| Pair(s) | Locus amplified | Template primer amplifies | Primer sequence (5’-3’) | |
| --- | --- | --- | --- | --- |
| 1, 2 & 3 | YBR295W | *S. cerevisiae* | F | GCGAACAGTACTCTAGAATG |
|  |  |  | R | CAGATTAAATTCAGCTTGC |
|  |  | *S. paradoxus* | F | GTAACTGGTAAGGGCGTAG |
|  |  |  | R | CAATTTCAAGACGAGCAG |
|  | YLL054C | *S. cerevisiae* | F | CATTGCATCTCAGAAAAAG |
|  |  |  | R | GGAGGATACAATTTGCTTG |
|  |  | *S. paradoxus* | F | GAAGAAACAACGGTGAAAAG |
|  |  |  | R | CCAGAGATCCGTTGAAATAC |
| 4 & 5 | YCR093W | *S. cerevisiae* | F | CATCGCAGGGATTAATAATG |
|  |  |  | R | GTAGCAGCTTCGACGATAG |
|  |  | *S. paradoxus* | F | GAGAAGGGCGTAATCAGTG |
|  |  |  | R | CTTGTCTGGTTTTGGAATC |
|  | YDL240W | *S. cerevisiae* | F | CAGTGATCAAGAATGCATTG |
|  |  |  | R | CAGTTCTTGATTTGACTAGGG |
|  |  | *S. paradoxus* | F | CCATTGATAGTAGTCCGACAG |
|  |  |  | R | CTTTATGGCGTAAATACAAG |

Table of the PCR primer sequences used to identify hybrid and non-hybrid zygotes. Two sets were used in each pairing to verify results. (F = Forward primer, R = Reverse primer)
